# AZD9773 is a novel and potent anti-TNFα polyclonal ovine immune Fab

**DOI:** 10.1186/cc9681

**Published:** 2011-03-11

**Authors:** P Newham, P Ceuppens, G Davies, J Growcott

**Affiliations:** 1 AstraZeneca, Macclesfield, UK

## Introduction

The release of cytokines into the circulation is an essential part of the inflammatory cascade that underlies sepsis. Experimental and clinical data have shown that the proinflammatory cytokine TNFα is a principal mediator of this cascade [1-3]. The investigational drug AZD9773, intended for intravenous infusion, contains ovine immune fragments (Fabs) of IgG that bind to human (hu)-TNFα. Here we describe the *in vitro *and *in vivo *pharmacology of AZD9773.

## Methods

AZD9773 binding to human TNFα was assessed using surface plasmon resonance (SPR) technology. AZD9773 functional potency was profiled versus recombinant human (r-hu)-TNFα and natural (WHO International Standard) (n)-TNFα in TNFα-mediated cytotoxicity assays using the L929 cell line. Finally, humanised mice (Tg1278/TNF^-/-^: hu-TNFα transgenic, murine TNFα null) were used to assess AZD9773 effects on endotoxin-induced serum cytokines, chemokines and related factors.

## Results

SPR assays revealed that r-hu-TNFα bound to immobilised AZD9773 total Fabs with an equilibrium dissociation constant (K_d_) of ~60 nM. AZD9773 neutralised both r-hu-TNFα and n-TNFα biological activity in the L929 cytotoxicity assays. AZD9773 neutralised r-hu-TNFα with an apparent inhibitory constant (K_i_) of approximately 40 pM. In humanised mice, AZD9773 produced a statistically significant reduction in 29 out of 60 serum cytokines and related factors (including hu-TNFα and murine IL-6).

## Conclusions

AZD9773 is a potent TNFα neutralising ovine immune Fab and, considering the modest AZD9773:TNFα binding affinity, these data indicate that there is significant synergy in neutralising TNFα bioactivity between the polyclonal anti-TNFα species that comprise AZD9773. The *in vivo *suppression of 29 out of 60 induced serum cytokines, chemokines and related factors confirms the significant role for TNFα in eliciting acute endotoxin responsiveness.
